# Evaluation of the impact of immediate versus WHO recommendations-guided antiretroviral therapy initiation on HIV incidence: the ANRS 12249 TasP (Treatment as Prevention) trial in Hlabisa sub-district, KwaZulu-Natal, South Africa: study protocol for a cluster randomised controlled trial

**DOI:** 10.1186/1745-6215-14-230

**Published:** 2013-07-23

**Authors:** Collins C Iwuji, Joanna Orne-Gliemann, Frank Tanser, Sylvie Boyer, Richard J Lessells, France Lert, John Imrie, Till Bärnighausen, Claire Rekacewicz, Brigitte Bazin, Marie-Louise Newell, François Dabis

**Affiliations:** 1 Africa Centre for Health and Population Studies, University of KwaZulu-Natal, Somkhele, KwaZulu-Natal, South Africa; 2 University Bordeaux, ISPED, Bordeaux, France; 3 INSERM, ISPED, Centre Inserm U897, Epidemiologie-Biostatistique, Bordeaux, France; 4 INSERM, UMR912 (SESSTIM), Marseille, France; 5 UMR-S912, IRD, Aix Marseille Université, Marseille, France; 6 ORS PACA, Observatoire Régional de la Santé Provence-Alpes-Côte d’Azur, Marseille, France; 7 Department of Clinical Research, London School of Hygiene and Tropical Medicine, London, UK; 8 INSERM U1018, CESP, Epidemiology of Occupational and Social Determinants of Health, Villejuif, France; 9 University of Versailles Saint-Quentin, UMRS 1018 Villejuif, Paris, France; 10 Centre for Sexual Health and HIV Research, Research Department of Infection and Population, Faculty of Population Health Sciences, University College London, London, UK; 11 Department of Global Health & Population, Harvard School of Public Health, Harvard University, Boston, USA; 12 Agence nationale de recherches sur le sida et les hépatites virales (ANRS), Paris, France; 13 Faculty of Medicine, University of Southampton, UK

**Keywords:** HIV infections, Antiretroviral therapy, Prevention, South Africa

## Abstract

**Background:**

Antiretroviral therapy (ART) suppresses HIV viral load in all body compartments and so limits the risk of HIV transmission. It has been suggested that ART not only contributes to preventing transmission at individual but potentially also at population level. This trial aims to evaluate the effect of ART initiated immediately after identification/diagnosis of HIV-infected individuals, regardless of CD4 count, on HIV incidence in the surrounding population. The primary outcome of the overall trial will be HIV incidence over two years. Secondary outcomes will include i) socio-behavioural outcomes (acceptability of repeat HIV counselling and testing, treatment acceptance and linkage to care, sexual partnerships and quality of life); ii) clinical outcomes (mortality and morbidity, retention into care, adherence to ART, virologic failure and acquired HIV drug resistance), iii) cost-effectiveness of the intervention. The first phase will specifically focus on the trial’s secondary outcomes.

**Methods/design:**

A cluster-randomised trial in 34 (2 × 17) clusters within a rural area of northern KwaZulu-Natal (South Africa), covering a total population of 34,000 inhabitants aged 16 years and above, of whom an estimated 27,200 would be HIV-uninfected at start of the trial. The first phase of the trial will include ten (2 × 5) clusters. Consecutive rounds of home-based HIV testing will be carried out. HIV-infected participants will be followed in dedicated trial clinics: in intervention clusters, they will be offered immediate ART initiation regardless of CD4 count and clinical stage; in control clusters they will be offered ART according to national treatment eligibility guidelines (CD4 <350 cells/μL, World Health Organisation stage 3 or 4 disease or multidrug-resistant/extensively drug-resistant tuberculosis). Following proof of acceptability and feasibility from the first phase, the trial will be rolled out to further clusters.

**Discussion:**

We aim to provide proof-of-principle evidence regarding the effectiveness of Treatment-as-Prevention in reducing HIV incidence at the population level. Data collected from the participants at home and in the clinics will inform understanding of socio-behavioural, economic and clinical impacts of the intervention as well as feasibility and generalizability.

**Trial registration:**

Clinicaltrials.gov: NCT01509508; South African Trial Register: DOH-27-0512-3974.

## Background

Thirty years after the discovery of the HIV, the pandemic does not show significant signs of abating [1]. HIV plasma viral load (VL) in the index HIV-infected individual is the dominant determinant of transmission to others, as shown in studies of heterosexual couples and mother/child pairs [2,3]. Antiretroviral therapy (ART) with fully suppressive antiretroviral (ARV) drug combinations lowers VL and substantially decreases the risk of transmission. The recent results from the HPTN 052 trial showed that ART reduced transmission by 96% in stable heterosexual couples where one partner was HIV-infected and the other not, and in which the partners had disclosed their HIV status to each other [4].

Following on from the HPTN052 trial, recent results from the annual population-based HIV surveys conducted in the Hlabisa sub-district, KwaZulu-Natal, South Africa (in the population immediately adjacent to the trial setting) demonstrated significant reductions in the risk of acquiring HIV in areas of high ART coverage [5]. The study showed that population-level reductions in the transmission of HIV can be achieved in nurse-led, devolved, public-sector programmes in rural sub-Saharan African settings where complete coverage of therapy under existing treatment guidelines has not yet been attained. However, it is not yet known what impact could be achieved on HIV incidence at a population level if ART was given to all HIV-positive individuals [6].

In 2009, results from a mathematical modelling exercise using a hypothetical population and assumptions relating to the South African (SA) setting suggested that ‘universal voluntary HIV testing and immediate ART (regardless of CD4 count) combined with present prevention approaches could have a major effect on severe generalised HIV epidemics’ [7]. Subsequent modelling confirmed that the impact of test-and-treat approaches on HIV incidence depends on the epidemiological context (characteristics of the sexual partner network, including heterogeneity, concurrency and mixing; HIV testing uptake; linkage to care and ART coverage; among others) [8-10]. Earlier treatment initiation may reduce HIV incidence at a population level and well-conducted observational studies have shown that it may likely benefit the individual [11,12]. While the question of when to start ART is still a matter of debate worldwide and is the subject of two ongoing randomised trials - the NIH-sponsored START trial (clinicaltrials.gov identifier NCT00867048) and the ANRS-sponsored TEMPRANO trial (clinicaltrials.gov identifier NCT00495651), recent evidence suggests long-term benefits of starting ART earlier would likely be of particular importance in settings where the incidence of life-threatening HIV-related diseases occurring at relatively high CD4 levels (tuberculosis, invasive bacterial diseases, and possibly malaria) is substantial, a typical situation in most of sub-Saharan Africa including SA [13].

We hypothesise that HIV testing of all members of a community, followed by immediate ART initiation of all HIV-infected adults regardless of immunological or clinical staging will reduce onward sexual transmission of HIV and hence HIV incidence. To test this hypothesis, we designed a cluster-randomised trial [14] for implementation in Hlabisa sub-district, KwaZulu-Natal, SA, in a two-phased approach. Enrolment in the first phase started in 2012, first in four clusters then in an additional six clusters in early 2013; if results from the first phase indicate good acceptability and feasibility, it is expected that the second phase will then be implemented in the other 24 (2 × 12) clusters from 2014 to 2016.

### Objectives

#### Main trial objective

The main objective is to evaluate the impact of ART, initiated immediately after identification/diagnosis of HIV-infected adults irrespective of CD4 count or clinical staging, on the incidence of new HIV infections in the general population over a period of 24 months, in comparison with treatment initiated according to national eligibility criteria.

#### Specific trial objectives

The objectives among all participants, to determine acceptability and feasibility over a 24-month period of providing repeat HIV testing to adult members of a community followed with immediate ART for HIV-infected individuals, and more specifically to compare between the two arms (intervention versus control) the following: acceptability and uptake of initial and repeat HIV counselling and testing; sexual behavioural changes and prevention practices at individual level; community attitudes toward HIV-infected individuals and perception of care and HIV treatment; and household expenditure, cost-effectiveness and other economic consequences of the trial at household level.

Among HIV-infected participants, to compare between the two arms over a 24-month period, the objectives are to determine: acceptability and uptake of HIV care and ART provided at the trial clinics; therapeutic success/effectiveness and tolerability of ART, participant retention in care, mortality and morbidity, tuberculosis notification rates, virological treatment failure, acquired HIV drug resistance and toxicity; adherence to ART; and quality of life.

Within the health system, the objective is to identify the challenges faced by the health care system and health care professionals in providing the trial intervention and coping with the increased number of trial participants.

### The aim of the first phase of the trial

The aim will be to inform the decision-making towards the implementation of the trial second phase through: i) the validation and updating of the model parameters (uptake of HIV testing, linkage to care upon HIV diagnosis, internal migration and ART initiation) used to estimate the trial sample size and HIV incidence; ii) assessment of the acceptability and feasibility of the intervention.

## Methods

### Study design

**Figure 1:**
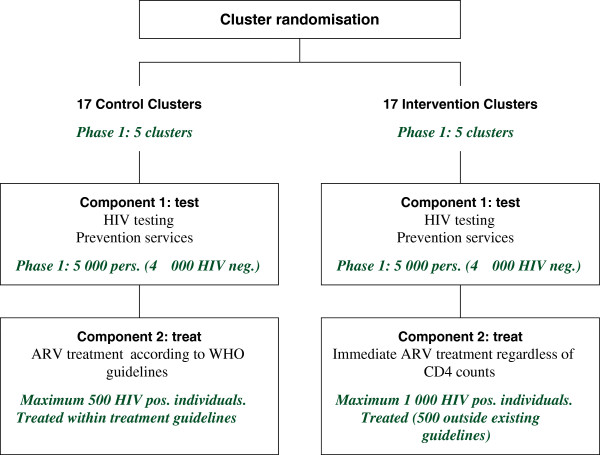
Description of the different components of the ANRS 12249 TasP trial.

This is a cluster-randomised controlled trial planned to take place over five years (2012 to 2016). The overall trial consists of 34 (2 × 17) clusters divided into two arms, covering a total population of 34,000 eligible inhabitants, of whom an estimated 27,200 would be HIV-uninfected at trial commencement. In the trial communities HIV testing of all members will take place at regular intervals (component 1). The impact of two different ART initiation strategies on HIV incidence (component 2) will then be compared as illustrated in Figure 1. During the first phase of the trial, three consecutive rounds of home-based HIV testing are implemented in the first four clusters; the duration of the first calendar round of testing (CR) is six months, the second and the third CRs are four months. Six-monthly CR is planned in the subsequent six clusters and for the rest of the clusters if the second phase is implemented. Follow up of HIV-infected participants in trial clinics (one in each cluster) is planned for 24 months in the overall trial, and for a maximum of 20 months during the first phase if the trial does not continue into the second phase.

### Setting

**Figure 2:**
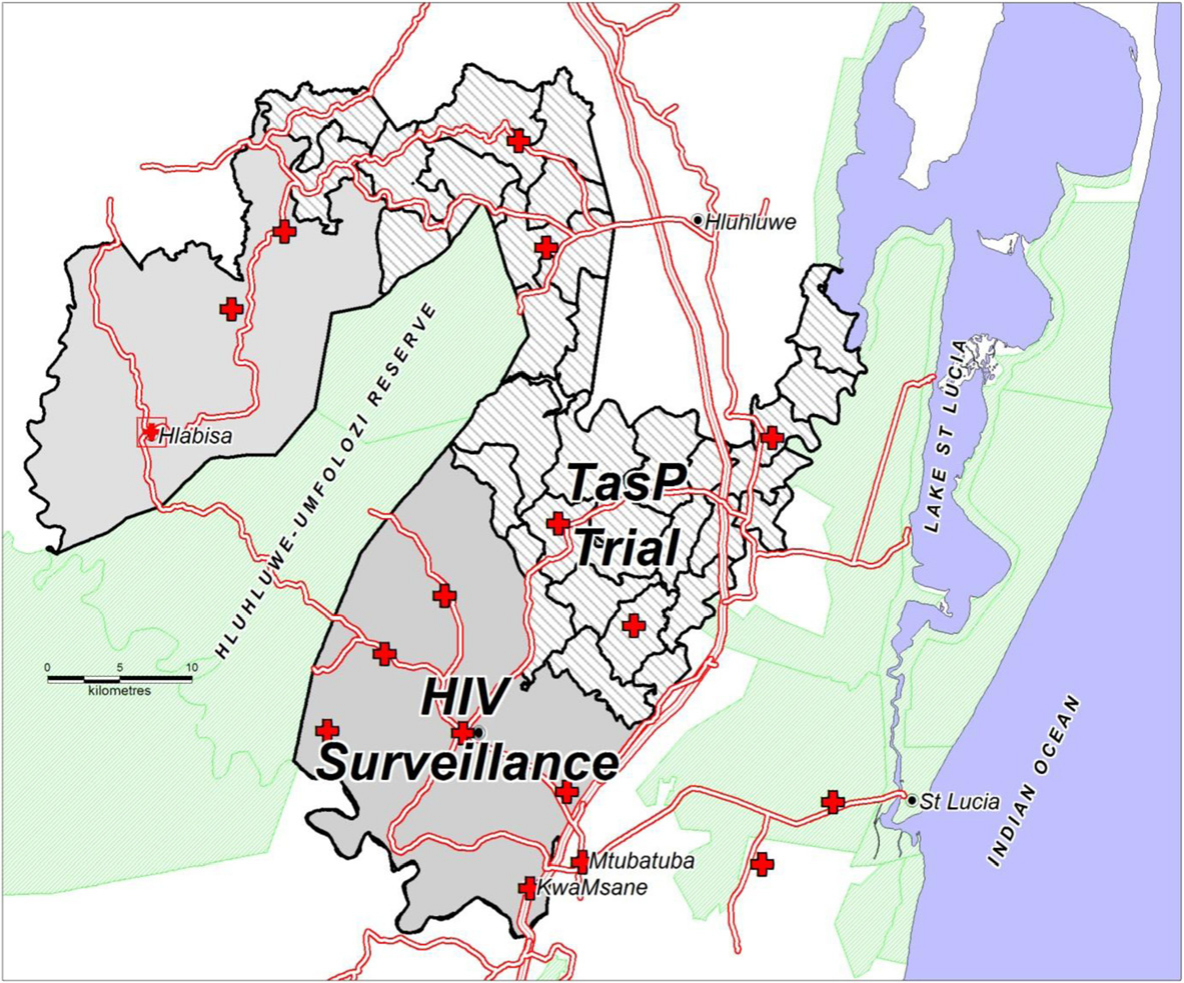
ANRS 12249 TasP Trial clusters within Hlabisa sub-district (South Africa).

The trial is conducted in Hlabisa sub-district (Figure 2), Umkhanyakude district, of northern KwaZulu-Natal, SA. This predominantly rural setting of 1,430 km^2^ in size has a population of approximately 220,000 Zulu-speaking people. In this sub-district, the Africa Centre for Health and Population studies, a research institute at the University of KwaZulu-Natal [http://www.africacentre.com] carries out sociodemographic and HIV surveillance and clinical research. Estimated HIV prevalence among adults in Hlabisa sub-district was 20% in 2007 [15], with considerable spatial heterogeneity [16,17]. The crude HIV incidence rate within the Africa Centre Demographic Surveillance Area (DSA), an area covering 400 km^2^ within the sub-district, was estimated at 3.4 per 100 person-years between 2003 and 2007 [18,19] declining to 2.9 per 100 person-years between 2004 and 2009 [20].

In 2004, the Africa Centre and the KwaZulu-Natal Department of Health (DoH) established the Hlabisa HIV Treatment and Care Programme, devolved to all 17 primary health care clinics in the sub-district [21]. HIV testing is available at all primary health care clinics and the district hospital, including mobile clinics regularly offering testing in communities, mobile testing clinics at community events, and home-based HIV counselling and testing (HCT) in households across the sub-district, which started in 2009 and has proved to be highly acceptable with a high uptake rate [22]. Comprehensive prevention services include information and education, condom promotion and distribution; medical male circumcision; syndromic management of sexually transmitted infections; post-exposure prophylaxis after sexual assault; and family planning. HIV treatment and care is provided free at all primary health care clinics, with ART eligibility determined by SA guidelines, which since August 2011 used a CD4 350 cells/μL eligibility cutoff for all adults. By the end of 2011, more than 40,000 individuals had accessed HIV care in the sub-district and over 20,000 of them had been initiated on ART. The ART coverage within the DSA was estimated at 37% of all HIV-infected adults [5].

### Trial participants (inclusion and exclusion criteria)

Individuals are eligible for trial participation if aged 16 years and above and a member of a household in the designated cluster (head of household defines membership status in Zulu culture). They must be able and willing to give written informed consent for trial participation and/or HCT. Individuals considered unable to provide informed consent will include those with severe uncontrolled psychiatric disorders, and those with neurological impairment resulting in an inability to participate in the informed consent process.

### Cluster design

The randomisation units within the TasP trial are geographic clusters within Hlabisa sub-district, outside of the Africa Centre DSA [23]. In brief, the trial area consists of 211 local areas (neighbourhoods). Clusters were designed to encompass social and sexual networks based on earlier studies in the DSA [24] with the aim of keeping the potential for cross-arm contamination to a minimum. The 211 local areas were thus aggregated into 48 clusters of between one and six contiguous neighbourhoods, comprising an average of 1,000 individuals aged 16 years and above and having a median area of 19 km^2^.

From a statistical efficiency perspective (smallest sample size) more clusters of fewer people should be used. However, having very small units of randomisation would result in greater potential for contamination. Adjacent neighbourhoods have thus been combined with this trade-off between statistical efficiency and contamination potential in mind in order to form relatively large randomisation units (with similar numbers of participants) with a distinct social identity. This reduces the risk of contamination whilst still retaining a relatively large number of clusters for the trial (Figure 2).

### Sample size calculation

We used two independent well-established mathematical models, the cost-effectiveness of preventing AIDS complications (CEPAC) [25], and a microsimulation model for decision support in sexually transmitted disease control (STDSIM) [26]) to demonstrate that a 30% reduction in cumulative HIV incidence (5% versus 3.5%) in HIV-negative participants over two years would be feasible across a range of parameter space. The models assumed that 10% of individuals in the trial would select a partner from a community randomised to the opposite arm of the trial. Sample size calculations indicated that 34 clusters (17 in each arm), with 1,000 consenting participants 16 years or older in each cluster (n = 34,000; 27,200 HIV-negative), were required to achieve this objective. This sample size has 88% power to detect this difference, with an alpha-type-one error of 5% (two-tailed) and an allowance of 20% of HIV-negative participants lost to follow up for the primary outcome measure. We assumed a coefficient of variation of 0.25 to account for within-group variation between clusters, the value of which was based on the values used in other randomised trials in Africa with HIV incidence as the outcome measure [27]. The cluster size variability correction was not included in the calculation because the clusters were designed to be of approximately equal population size.

The first phase is conducted on approximately 10,000 participants in 10 clusters. This sample size allows the measurement of the proportion agreeing to test over three rounds of home-based HIV testing to within 1% (95% CI).

### Randomisation

Randomisation was performed by the trial statisticians before the start of the first phase. Communities were randomly allocated in equal measure to control and intervention communities (17:17). To minimise the degree of between-cluster variation, communities were stratified on the basis of predicted HIV prevalence, using HIV surveillance data from the Africa Centre’s DSA and data from antenatal clinics (six strata). Random number generation and the randomisation procedure were performed in MapInfo version 11.0. Randomisation was carried out within each stratum to derive an equal number of control and intervention communities per stratum.

#### Contamination minimisation

An important component of trial design has been the minimisation of contamination and the extent to which individuals will select their partner from an area randomised to the opposite arm of the trial. We have previously shown that partner choice in this population has a strong local geographical dimension [28]. As stated above, we allowed for 10% of individuals to have a partner from an area randomised to the opposite arm of the trial. In addition, in the randomisation, chance geographical groupings of communities randomised to the same arm of the trial occur and thus super-clusters are formed. In multiple simulations we showed that these super-clusters have a median population size of approximately 4,000. Given the well-documented geography of sexual partner choice in this population [28], these larger chance geographical groupings of clusters randomised to the same arm of the trial further reduce the potential for inter-arm contamination in the trial.

Another source of contamination, arguably more likely, refers to HIV-infected individuals within a control cluster breaking the assignment and seeking ART in an intervention cluster. We contain the threat of this type of contamination because only people registered through the household HCT as being eligible for immediate ART initiation will be able to do so within the trial, and data collection always starts in the household. To be initiated on ART at the trial clinic, individuals have to present their TasP card (given to them during the home-based HIV testing) and South African identity card to the clinic staff, who confirm from the netbook registration (as described below) whether or not a person is resident in an intervention cluster.

For the pilot phase, the initial four (2 × 2) clusters were selected on the basis of proximity to the Africa Centre. The pilot clusters all contain similar rural populations. In addition, each cluster pair (intervention/control) contained a local department of health clinic. The additional six (3 × 2) clusters were chosen to be contiguous to these initial four clusters.

### Study procedures

#### Home-based procedures

In 1999 (as part of the establishment of the Africa Centre) all homesteads in the Hlabisa sub-district were mapped and assigned an external bounded structure identification number (BSID), with details of the head of household at the time also recorded. Before the start of the TasP trial, these maps were compared with a recent satellite image of the sub-district, which allowed the Africa Centre geographic information system team to map homesteads newly constructed after 1999, before TasP trial fieldwork commences in any given cluster. Each fieldworker/counsellor carries a netbook with global positioning system (GPS) functionality and a map of the trial clusters showing a satellite image of the various homesteads and their BSIDs as well as the cluster boundaries. These netbooks are also equipped with software created at the Africa Centre for registering/processing homesteads and household members. The fieldworkers identify homesteads using the GPS function in the netbook, and seek permission from the head of household to enter the household. They enumerate all eligible members within the household, with specific information sheets for all individuals. A private space is identified and all individual adult household members are invited to give written permission to 1) complete an individual questionnaire, with or without anonymous sampling of blood for HIV surveillance, and/or 2) undergo confidential HCT. People who do not want to be tested for HIV in the household are informed they can attend any of the local clinics or the trial clinic for testing. Participants who give consent to anonymous HIV surveillance have blood collected by field workers by finger prick and stored on filter paper as dried blood spots (DBS) - DBS are stored at the Africa Centre virology laboratory in Durban. Basic demographic data are obtained from the household head for participants who decline both HCT and completion of the questionnaires. The same procedure applies in subsequent rounds of HIV testing.

#### HCT procedures and prevention services

HIV testing services provided in both trial arms consist of the current range of community and clinic testing options plus the implementation of regular rounds of home-based HIV testing, to achieve near-universal HIV testing coverage in the area and increase repeat HIV testing. During each HIV testing round, individuals providing written informed consent for HIV counselling and testing receive pre-test HIV counselling privately and confidentially, delivered by a trained counsellor. Rapid HIV testing is performed and test results are provided approximately 20 minutes after testing. The HIV status of the participant is documented in the fieldworkers’ netbooks.

The individual post-test HIV counselling session takes place as per routine DoH procedures, covering the prevention of acquisition of HIV for HIV-negative people and the implications of HIV infection for HIV-positive people. All participants have access to all available HIV prevention services in the routine health care system and these are also available at the trial clinics.

At the end of each working day, all fieldwork netbooks are handed to the Data and Specimen coordinator at the Africa Centre who synchronises the information from the netbooks to the main database. The information in the database is subsequently downloaded to all the netbooks used in the field, as well as in the clinics (see below). Hence, the netbooks come in from the field with information from different participants but leave the Africa Centre with identical information on all participants that have ever been registered in the trial and their trial status. This data synchronisation process makes it possible for the counsellor in the trial clinic to know exactly why someone bearing the TasP card attends the clinic.

#### Referral procedures to trial clinic

At the end of the HCT session, all participants are given a TasP referral/prevention card containing information about the trial clinics and the available services. Each participant is asked to take their TasP card with them when they attend the trial clinics, to identify them as a trial participant. A fingerprint is taken at the clinic during a participant’s first visit and confirmed at subsequent visits for correct identification and service eligibility (that is, treatment and care, prevention or HCT). Results from the clinic baseline VL test confirm HIV-positive status, except for individuals already established on ART. Where there is discrepancy in results between the VL test and the rapid antibody test, the DBS from the home-based testing stored in the Africa Centre virology laboratory can be accessed for confirmatory antibody testing.

Although all HIV-positive individuals (newly diagnosed and those with known positive status) identified by the fieldworkers are referred to the trial clinics, those who know their HIV status and are already in care have the option of continuing to receive care and treatment with their existing provider; they are invited to attend for review at the trial facility every six months to record clinical outcome data relating to the secondary outcomes. There is also ethics approval to link the Hlabisa HIV Treatment and Care Programme database (ARTemis) maintained by the Africa Centre with that of the trial database (also held and maintained at the Centre) using a participant’s unique South African identification number or name, if both first and last name matched, to obtain a more complete picture of linkage to care and treatment data of participants enrolled in the trial but opting to continue their HIV care with their current provider.

Participants who are not linked to care within three months of referral are followed up with a phone call and if necessary a repeat home visit is performed by a tracker to explore barriers to linkage as well as to support the participant in engaging with care.

#### Procedures for the management and care of HIV-infected participants

Trial clinics (one per cluster) are situated in close proximity to homesteads (<45 minutes walking distance for all participants within the cluster). These trial clinics are staffed by a counsellor and a nurse. A trial physician is available weekly at each clinic and on call at other times.

#### Baseline clinic visit

All participants identified as HIV-infected during any of the home-based HCT rounds are referred to their trial clinic for immediate assessment (within two weeks of diagnosis or as soon as feasible thereafter). The participants present their TasP card to the clinic team who search for the participant’s detail in the netbook to ensure they had completed the household procedures and they are from the appropriate cluster. They are provided standard counselling/ART education and adherence sessions by the ART counsellor and nurse, over one to three visits, as per DoH guidelines. They are asked to provide written consent to: 1) provide self-reported information and blood specimens for VL testing and 2) receive care and/or treatment at the trial clinics as per local DoH standards.

All HIV-infected participants consenting to treatment undergo clinical evaluation (Table 1) and in addition a CD4 point of care assessment (using Alere PIMA™ device tool, Alere Inc., Waltham, MA, USA). Patients are also interviewed by the counsellor on their HIV testing experience, ART perception, disclosure and economic situations.

**Table 1:** Follow-up calendar for HIV-infected participants eligible for ART

	**D0**/**D15**	**M0**	**M1**	**M2**	**M3**	**M4**	**M5**	**M6**	**M7**	**M8**	**M9**	**M10**	**M11**	**M12**	**M13**	**M14**	**M15**	**M16**	**M17**	**M18**	**M19**	**M20**	**M21**	**M22**	**M23**	**M24**
Consent	X																									
Medical history	X																									
**Nurse**																										
Physical examination	X		X	X	X	X	X	X	X	X	X	X	X	X	X	X	X	X	X	X	X	X	X	X	X	X
Weight, height	X				X			X			X			X			X			X			X			X
WHO clinical staging	X		X	X	X	X	X	X	X	X	X	X	X	X	X	X	X	X	X	X	X	X	X	X	X	X
Morbidity/hospitalisation	X		X	X	X	X	X	X	X	X	X	X	X	X	X	X	X	X	X	X	X	X	X	X	X	X
TB/STI screening if appropriate	X		X	X	X	X	X	X	X	X	X	X	X	X	X	X	X	X	X	X	X	X	X	X	X	X
CD4 point of care	X							X						X						X						X
ART initation		X						X						X						X						X
Adherence monitoring			X	X	X	X	X	X	X	X	X	X	X	X	X	X	X	X	X	X	X	X	X	X	X	X
**Laboratory**																										
HIV VL	X				X			X						X						X						X
Genotyping	As clinically indicated, in case of confirmed virological failure																									
CD4 counts	X							X						X						X						X
Haematology	X							X						X						X						X
Biochemistry	X				X			X						X						X						X
HBsAg	X																									
Beta hCG	X				X			X						X						X						X
Urinalysis	X				X			X						X						X						X
Plasma storage (−80°C)	X				X			X						X						X						X
Blood volume (mL)																										
**Questionnaires**																										
History of HIV infection		X																								
ART perception		X																								
ART knowledge		X						X						X						X						X
Disclosure and couple					X			X			X			X			X			X			X			X
Social and community support		X			X			X			X			X			X			X			X			X
Stigma and discrimination		X						X						X						X						X
Self-reported adherence					X			X			X			X			X			X			X			X
Health expenditure		X			X			X			X			X			X			X			X			X
Economic situation		X			X			X			X			X			X			X			X			X
Sexual behaviour		X			X			X			X			X			X			X			X			X
Quality of life								X						X						X						X
Satisfaction with care								X						X						X						X

ANRS 12249 TasP trial, *WHO* World Health Organisation, *TB* Tuberculosis, *STI* Sexually transmitted infections, *ART* Anti-retroviral treatment, *VL* Viral load, *HBsAg* Hepatitis B surface antigen, *Beta hCG* Beta human chorionic gonadotrophin.

All participants eligible for ART are seen within two weeks of this baseline visit, to review results of baseline investigation and, if indicated, initiate treatment as follows. In the intervention clusters, all HIV-infected participants are eligible for ART regardless of CD4 count and clinical stage. In the control clusters, HIV-infected participants are eligible for ART as per the 2011 SA and 2010 WHO guidelines [29]: CD4 count ≤350 cells/mm^3^ irrespective of clinical symptoms; WHO clinical stage 3 or 4 irrespective of CD4 count; and multidrug-resistant (MDR) or extensively drug-resistant (XDR) tuberculosis.

In the control clusters, patients not yet eligible for ART have a blood sample collected for baseline VL measurement and storage at the Africa Centre virology laboratory; they are invited to return to the study clinic in 4 to 6 months for repeat clinical assessment and CD4 count measurement (Table 2).

**Table 2:** **Follow**-**up calendar of HIV**-**infected participants not eligible for ART**

	**Month**				
	**0**	**6**	**12**	**18**	**24**
Consent	x				
Medical history	x				
**Nurse**					
Physical examination	x	x	x	x	x
Weight, height	x	x	x	x	x
WHO clinical staging	x	x	x	x	x
Morbidity/hospitalisation	x	x	x	x	x
TB/STI screening if appropriate	x	x	x	x	x
CD4 point of care	x	x	x	x	x
**Laboratory**					
Beta human chorionic gonadotropin	x	x	x	x	x
**Clinic**-**based survey**					
ART perception	x				
ART knowledge	x	x	x	x	x
Disclosure and couple	x	x	x	x	x
Social and community support	x	x	x	x	x
Stigma and discrimination	x	x	x	x	x
Health expenditure	x	x	x	x	x
Economic situation	x	x	x	x	x
Sexual behaviour		x	x	x	x
Depression and Anxiety		x	x	x	x
HIV quality of life		x	x	x	x
Satisfaction with care		x	x	x	x

ANRS 12249 TasP trial. *WHO* World Health Organisation, *TB* Tuberculosis, *STI* Sexually transmitted infections, *ART* Anti-retroviral treatment, *VL* Viral load.

In both clusters, patients declining ART are asked to consent to 6-monthly clinical assessment. Also in both clusters, participants already established on ART in the Hlabisa HIV Treatment and Care Programme (or any other public or private care provider) are encouraged to transfer their care to the trial clinics. Participants choosing to continue follow up from their normal provider are asked to consent to 6-monthly reviews and to provide permission for additional clinical information obtained from their provider to be used in the trial.

#### ART initiation visits and ARV drugs used within the trial

ART initiation takes place at month 0 (M0), ideally within four weeks of HIV testing or two weeks of enrolment at the trial clinic, unless purposely delayed for clinical reasons (for example, tuberculosis treatment). Once the decision to initiate ART has been made, patients receive ART and are followed up as long as the trial first phase continues and for a minimum of 24 months within the overall trial.

All ART drugs used in the trial are included in those recommended by the South African National Department of Health Adult HIV management guidelines. The standard first-line drug regimen for HIV-infected participants in both arms is the fixed drug combination (FDC) tenofovir (TDF 245 mg) + emtricitabine (FTC 200 mg) + efavirenz (EFV 600 mg) formulated as Atripla® (dose one tablet once daily). Use of other regimens is according to clinical indication [14].

#### Patient follow up

Participants receiving ART undergo monthly clinical evaluation as described in Table 1. Participants who are not on ART (not yet eligible for treatment or declining treatment) are referred to the trial nurse for pre-ART care, positive prevention services and 6-monthly clinical assessments (Table 2). Participants on ART who prefer to remain within the Hlabisa HIV Treatment and Care Programme are asked to consent to assessment by the trial nurse as per trial protocol (a trial nurse attends each of the DoH clinics once a week). The relevant data are collected from all clinic attendees irrespective of ART status, to complete the trial case report forms (CRFs). All participants followed in the trial clinics also complete a follow-up questionnaire administered by an independent interviewer to assess, among others, evolving perceptions of ART, stigma and discrimination, self-reported adherence, quality of life and health expenditure.

Participants missing a trial clinic appointment are phoned, and if contacted a new appointment is scheduled taking into account their drug supply. The names and details of those not reached are handed over to a tracker for tracing. One of the consent forms signed by the participants allows a member of the trial team to track them should they default. This is also the standard of care in the Hlabisa HIV Treatment and Care Programme. Participants are allowed to exit the clinic if they migrate out of the clusters but can attend another trial clinic if migration is to another cluster within the same arm of the trial. Those who migrate out of the clusters can also rejoin the trial clinic if they move back into the trial clusters.

#### Toxicity (adverse events) and treatment failure

In the case of toxicity or intolerance, single drug substitutions are allowed, as per SA guidelines [30]. The toxicities specifically monitored include renal dysfunction (TDF), liver function (nevirapine - NVP), and anaemia (zidovudine - AZT).

Routine virological monitoring occurs at 3 and 6 months, and then 6-monthly during follow up. The decision to switch to second-line ART is taken on the basis of two consecutive HIV VL measurements >1,000 copies/mL at least three months apart (as per current SA national guidelines) [30]. Information from genotyping is also made available in real time to the trial clinician to guide the decision whether to switch regimens. The standard second-line regimen is zidovudine (AZT) + lamivudine (3TC) + lopinavir/ritonavir (LPV/r). Participants positive for hepatitis B surface antigen (HBsAg) continue TDF in their ART regimen should they need to switch to second-line therapy. Trial participants fulfilling criteria for immunological and/or clinical failure in the absence of criteria for virological failure are reviewed by the trial clinician before any switch in drug regimen.

#### Care of patients at the end of the trial

Trial participants receiving ART at the end of the trial will be transferred into the Hlabisa HIV Treatment and Care Programme and will remain on the same drugs. The drugs will then be provided by the KwaZulu-Natal DoH, whether first-line or second-line drug regimens.

#### Concomitant therapies

Isoniazid preventive therapy, cotrimoxazole and multivitamins are provided to HIV-infected participants in the intervention and control clusters as per national policies at the time of the trial.

### Data collection tools

A combination of quantitative and qualitative trial instruments are used (Table 1) [14]. Home-based questionnaires will be completed at household level: head of households are interviewed on household assets, household income, food security. Home-based questionnaires are also completed at individual level, for all participants (core questions repeated at each round and a set of additional questions specific to certain rounds): individual-level social and demographic characteristics, HIV testing behaviour, sexual behaviour, prevention behaviours adopted (condom use, circumcision status), partnerships and sexual network patterns, attitudes and beliefs about HIV infection, HIV testing and treatment, stigma and disclosure, healthcare use and healthcare expenditures, and quality of life.

CRFs are completed by the counsellors at baseline and follow up, and a combined history and clinical examination CRF is completed by the research nurses, for all HIV-infected participants receiving care and treatment in the trials clinics irrespective of their ART status at enrolment into the trial and during follow up. A clinic-based survey will be performed for HIV-infected patients followed up at the trial clinics: testing experience (baseline only), ART knowledge, ART perception and decision, disclosure, sexual partnership details, sexual behaviour, self-reported ART adherence, HIV quality of life, stigma and discrimination, social and community support, economic and social outcomes, health expenditures, and satisfaction with care.

Clinic activity reports will be completed: monthly activity records of the trial clinics, patient waiting times, staffing levels, stock outage, the adequacy of trial logistics and support. Qualitative data will be collected to address the acceptability of repeat testing, adherence and quality of life among those starting treatment in the trial clinics: consumer advisory panels in each of the four original clusters to monitor the community experiences of the trial, perceptions and understanding of the intervention and early advice on any possible problems.

Repeat in-depth interviews will be performed to explore the adherence and quality of life in HIV-infected participants initiating ART in the trial clinics.

### Trial outcomes

#### Primary outcome

The primary outcome is HIV incidence measured at 24 months through repeat longitudinal HIV testing of blood samples collected on DBS during the home visits.

#### Main secondary outcomes

##### Among all participants

The acceptability and uptake of HIV testing will be assessed as the proportion of participants who are tested for HIV among all those eligible, among those never tested and among those testing HIV-negative at the first round and who accept repeat HIV testing. We will also assess the proportion of participants who know their HIV status and who disclose their HIV status. These outcomes will be calculated for each successive round of home-based HIV testing and will be based on data collected during the home visits. HIV testing history outside the trial, and perception about repeat HIV-testing will also be studied.

Behavioural changes at individual level will be studied through the number of sexual partnerships, prevention behaviours adopted (for example, condom use and HIV testing outside the trial, circumcision, contraceptive use) and attitudes towards HIV-infected individuals. These data will be collected from all enumerated participants during successive rounds of home-based testing. This will facilitate longitudinal as well as repeat cross-sectional analyses regardless of participants’ self-reported HIV status.

Individual perceptions of community awareness, and individual attitudes and behaviours towards HIV, repeat HIV testing and ART will be measured during the successive rounds of home-based testing.

Cost-effectiveness analysis, assessing the number of life years gained and the number of quality-adjusted life years (QALYs) saved, will be carried out based on data from the TasP trial and will be completed with a mathematical Markov model, comparing treatment initiation as soon as HIV infection is diagnosed to delayed treatment initiation according to WHO recommendations.

#### Among HIV-infected participants

The following outcomes will be measured for participants newly diagnosed with HIV infection and those known to be infected but not receiving ART.

Acceptability and uptake of care and ART will be assessed using time from receipt of HIV test result to enrolment at the trial clinics or primary health care clinics, transfer of care of patients currently being monitored (as yet ineligible for ART) in the primary health care clinics to the trial clinics; characterisation of the population entering into treatment and care and evolution of the social factors that facilitate entry (for example, household composition, social support, disclosure to family, treatment knowledge). All of these intermediate measures will be useful in determining the need and content of any special interventions to support treatment uptake in the main trial.

The therapeutic success and evaluation of ART will be estimated as the proportion of HIV-infected participants (ART-naïve at enrolment) with VL <50 copies/mL after six months of ART.

Additional secondary outcomes will be explored in all HIV-infected participants including those already receiving ART at enrolment as follows. Retention in care, defined as those still under active follow up in the trial, will be assessed at 12 months and 24 months post- enrolment. Loss to follow-up on ART will be defined as ≥3 consecutive missed appointments. For those not eligible for ART, loss to follow-up in control clusters will be defined as >9 months from last clinic visit and/or CD4 cell count. Mortality, severe morbidity including WHO stage IV disease, serious non-AIDS events, tuberculosis and grade 3 and 4 adverse events (clinical and laboratory) will be documented. Virological failure will be defined as two consecutive VL >1,000 copies/mL measured 3 months apart. Adherence will be measured using 1) a scale that has already been translated and validated by Africa Centre researchers with patients from the Hlabisa Treatment and Care Programme, which uses a combination of a visual analogue scale, pill identification test and pill count [31] and 2) an additional scale constructed to limit both recall and social desirability bias and which has been tested in different settings [32-34]. Quality of life will be assessed using the Patient Reported Outcomes Quality Of Life specific to HIV (PROQOL-HIV) [35,36] and the HIV/AIDS stigma instrument for PLWHA (HASI-P) [37].

To assess drug resistance, HIV-1 genotypic resistance testing will be performed for HIV-infected individuals identified with virological failure during the trial, HIV-infected individuals already on ART at trial entry and identified with virological failure at enrolment and all participants who seroconvert within the trial. We will analyse all virological sequences using a phylodynamics framework. Phylodynamics can be used to estimate the date of origin of epidemiologically important events such as the introduction of a new viral strain in a geographical area and identification of transmission networks within and between clusters.

### Analysis

The primary outcome is incidence of HIV-1 infection, measured using DBS collected during home-based HCT, and defined by seroconversion from negative to positive between HCT rounds. An intention-to-treat analysis based on the randomised clusters will be conducted for this primary trial outcome. Incidence rates per 100 person-years will be calculated for the whole follow-up period. Adjusted HIV incidence rate ratios in the intervention group relative to the control group will be based on a multi-level Poisson regression taking into account the intra-cluster correlation and the repeated measurements when needed.

We will perform analyses using cluster-level approaches as robustness analyses for checking consistency of the results. However, we will focus on Poisson regression (including random effects) for the main outcome (HIV incidence) and logistic regression with the generalised estimated equation (GEE) approach for binary outcomes, thus allowing for both levels (individual and cluster levels) with easy adjustment for confounding factors [38]. As the method does not perform well for a low number of clusters, we plan to use the correction (of the sandwich estimator and statistics distribution i.e. t-distribution) proposed by Mancl and Derouen [39], which is particularly appropriate when the cluster size does not vary.

Because of the possibility that the randomisation may lead to some imbalance in the distribution of HIV risk factors across the trial arms, an adjustment for baseline characteristics known to be associated with HIV transmission risk in this population will be undertaken. These include HIV prevalence of the cluster, age, sex, marital status, education level attained and migration status.

With regard to the randomisation, strata will be defined on the basis of predicted HIV prevalence. Clusters will be stratified according to 5% prevalence intervals (<15, 15 to 20, 20 to 25, >25). With regard to the sample size calculation, clustering was taken into account by the coefficient of variation (0.25), the value of which was based on the values used in other randomised trials in Africa with HIV incidence as the outcome measure [27]. This coefficient of variation is necessarily conservative. For instance, a coefficient of variation of 0.2 would have led to the need for a population of 30,000 (24 clusters). Through stratifying on the basis of estimated HIV prevalence we expect to minimize inter-arm variation in HIV incidence. The sample size calculation is very conservative and will be further adjusted on the basis of the results of the first phase of the trial. In addition, the extensive data related to population movements and partnership patterns that will be collected over the course of the trial will be used in a parallel set of secondary analyses to identify risk factors that influenced the risk of transmission. If the intervention does not have a significant impact, these important analyses will help to identify which factors were likely to have led to the trial result.

### Criteria for continuation/discontinuation at the end of the first phase

The following criteria would be grounds for not continuing from the first to the second phase. Feasibility: clear indication that given the parameters measured during the first phase (HIV prevalence in the clusters at baseline and assessment of incidence; initial HIV testing uptake; repeated HIV testing uptake; ART uptake; migration and the extent of sexual partnerships with people outside the trial setting), the TasP trial will lack the statistical power to detect significant HIV incidence differences between the intervention and control communities. Acceptability: clear indication from the clinic-based survey and the qualitative in-depth interviews conducted during the first phase that the TasP approach is not acceptable in our setting, in terms of community attitudes and beliefs about HIV, HIV counselling and testing, stigma and disclosure, participation in the TasP trial, and the acceptability of ART for the benefit of other community members. The DSMB (see Additional file 1) will be able to consider additional parameters measured during the first phase, such as tuberculosis incidence, or information from other studies.

## Discussion

Two meta-analyses of observational studies [40,41] and a recently concluded randomised controlled trial [4] provided evidence that antiretroviral treatment is effective in preventing sexual transmission of HIV from the index partner to the uninfected one. ART is now recommended by the WHO to prevent HIV transmission in sero-discordant couples irrespective of whether the index partner requires ART for their own health or not [42]. However, the question remains whether the effectiveness of ART in preventing transmissions at the individual level can be replicated at the population level.

A population-based cohort study conducted by the Africa Centre team examined the impact of ART coverage on HIV incidence at the population level [5]. This study showed a decrease in the risk of HIV acquisition when ART coverage in the immediately surrounding area exceeds 20% of the HIV-infected population. The authors recommended that cluster randomised trials at population level are needed to confirm the validity and generalizability of their findings.

Randomised controlled trials are the gold standard for establishing the efficacy of a health intervention. Evaluating the impact of the TasP package of services on HIV incidence does not focus on the HIV-infected individuals who receive the intervention, but rather their HIV-uninfected sexual contacts. Had we opted for conventional individual randomization of individuals, it would have necessitated tracing all their sexual contacts, an option which is not realistic in this setting. A less sophisticated approach would have consisted of a simple comparison of HIV incidence, before and after expansion of an ART programme, similar in approach to the recent paper from our group. However, many other factors besides introduction of ART may change from "before" to "after", introducing bias and incorrect attributions of causality to any observed change in incidence. Hence we have adopted a cluster-randomised trial approach as the best design to examine the effectiveness of the TasP intervention in reducing HIV incidence at the population level.

Within the TasP trial, HIV-infected patients residing in the intervention clusters are encouraged to start ART irrespective of their CD4 count level; current SA eligibility criteria recommends ART initiation at a CD4 count threshold of 350 cells/µL or below. However, increasingly treatment guidelines are moving towards earlier initiation of therapy [43], which is driven predominantly by evidence from observational studies.

The development of HIV drug resistant strains is a concern in situations where treatment is started at higher CD4 count thresholds, as in the case of ART as prevention, since people will be on HIV treatment for longer with increased potential for of sub-optimal adherence. Some studies have shown that adherence is lower in individuals starting ART at higher CD4 counts [44,45]. However, limited available data do not suggest an increased risk of drug resistance with earlier HIV treatment [46]. Our trial will be a valuable addition to knowledge in this regard.

In the TasP trial, quantitative and qualitative social science research is implemented at each stage to inform understanding of the social determinants of the intervention, uptake at the individual and community-level, and the social, economic and behavioural consequences at individual, household and community level. However, such social science evidence alone cannot guide decisions on scaling-up TasP strategies in public health programmes. If TasP is demonstrated to be efficacious, new questions will need to be addressed to inform public health and operational policies. These questions fall under three broad headings: firstly, what are the social and behavioural consequences of large numbers of people knowing their HIV-status and potentially beginning ART early? Will the impacts on sexual behaviour, disclosure and stigma be positive or harmful both in the short term and in the long term? What will be the impact on marginalised key populations? Secondly, does seeing more healthy people with HIV attending clinics alter community perceptions of disease and care, and if that is the case, can salient positive changes be identified and replicated in other settings? Thirdly, what are the operational and ethical implications of transforming research interventions into routine care? What are the requirements for sustainability? Which new ethical guidelines will be needed to protect the most vulnerable populations?

Our trial documenting very important issues such as HIV testing uptake, linkage to care, retention in care and adherence to therapy and the social context in which these occur will provide valuable information about the feasibility and acceptability of universal initiation of ART both for individual and public health benefits. Cost effectiveness data will also inform decisions on whether and how to take such strategies to scale or to invest in existing proven cost-effective interventions such as male medical circumcision and expanding coverage at current ART initiation thresholds [47]. There is a window of opportunity to try and provide answers to these important questions before clinical practice overtakes evidence-based policy.

### Trial oversight

The Steering Committee (SC) will be responsible for the conduct of the trial and its overall organization. The SC is the trial decision body, for all scientific and administrative aspects. The SC will be co-chaired by the Principal Investigators. It will comprise the team investigators in South Africa, France and Switzerland and representatives of the Sponsor. The SC will meet as regularly as needed on conference calls and face-to-face meetings. The SC ensures the correct implementation of the study and compliance with the protocol, and verifies its ethical compliance. It decides about any relevant changes to the protocol, necessary for continuation of the study.

The Scientific Advisory Board (see Additional file 1) would oversee the overall conduct of the trial. Its mission is to make sure and to report, particularly to the Sponsor, whether the study is carried out properly scientifically, ethically and logistically.

The DSMB would monitor the main safety and efficacy outcome measures and the overall conduct of the trial, with the aim of protecting the safety and the interests of the trial participants. The DSMB would meet on a regular basis during the trial and at least once a year.

### Ethical safeguards

This trial was approved by the Biomedical Research Ethics Committee of the University of KwaZulu-Natal on 2 February 2012 for the control clusters and on 6 July 2012 for the full trial. The trial is being conducted with the permission of the KwaZulu-Natal DoH, South Africa. Permission by the South African Medicines Control Council was granted on 28 June 2012. Consent for the collection of data and the retention of residual samples will be solicited from individuals following established procedures in the Africa Centre surveillance, and approved by the University of KwaZulu-Natal Biomedical Research Ethics Committee.

Routine community road shows will be used to ensure continuous feedback between the investigators and the communities.

## Trial status

The trial started enrolling participants in March 2012.

## Abbreviations

ANRS: Agence nationale de recherches sur le sida et les hépatites virales; ART: Antiretroviral treatment; ARV: Antiretroviral drugs; AZT: Zidovudine; beta hCG: Beta human chorionic gonadotrophin; BSID: Bounded structure identification number; CR: Calendar round; CRF: Case report form; DBS: Dried blood spot; DoH: Department of Health; DSA: District surveillance area; DSMB: Data Safety and Monitoring Board; EFV: Efavirenz; FDC: Fixed drug combination; FTC: Emtricitabine; GEE: Generalized estimated equation; GPS: Global positioning system; HBsAg: Hepatitis B surface antigen; HCT: HIV counselling and testing; LPV/r: Lopinavir/ritonavir; MDR: Multidrug resistant; NVP: Nevirapine; PLWHA: (HASI-P) HIV/AIDS Stigma Instrument; PROQOL-HIV: Patient reported outcomes quality of life specific to HIV; QALY: Quality-adjusted life year; SA: South Africa; STI: Sexually transmitted infection; TasP: Treatment as prevention; TB: Tuberculosis; 3TC: Lamivudine; TDF: Tenofovir; VL: Viral load; WHO: World Health Organisation; XDR: Extensively drug resistant.

## Competing interests

The authors declared that they have no competing interests.

## Authors' contributions

FD and MLN are the principal investigators and supervised the trial protocol development. CI (South Africa) and JOG (France) are the trial coordinators. FT is responsible for the statistical trial design. CI and JOG wrote the initial draft of the paper. FT, SB, RL, FL, JI, TB, CR and BB contributed to writing and editing the paper. All members of the TasP Working Group participated in designing specific aspects of the trial and approved this submitted version of the manuscript. All authors read and approve the final manuscript.

## Supplementary Material

Additional file 1 Table S1 Composition of the TasP Study Group. Click here for file

## References

[B1] UNAIDS Report on the Global AIDS Epidemic 2012 Geneva: UNAIDS [http://www.unaids.org/en/media/unaids/contentassets/documents/epidemiology/2012/gr2012/20121120_UNAIDS_Global_Report_2012_en.pdf]. Accessed 18 January 2013

[B2] Quinn TC Wawer MJ Sewankambo N Serwadda D Li C Wabwire-Mangen F Meehan MO Lutalo T Gray RH Viral load and heterosexual transmission of human immunodeficiency virus type 1. Rakai Project Study Group N Engl J Med 2000 342 921 929 10.1056/NEJM200003303421303 10738050

[B3] Garcia PM Kalish LA Pitt J Minkoff H Quinn TC Burchett SK Kornegay J Jackson B Moye J Hanson C Zorrilla C Lew JF Maternal levels of plasma human immunodeficiency virus type 1 RNA and the risk of perinatal transmission. Women and Infants Transmission Study Group N Engl J Med 1999 341 394 402 10.1056/NEJM199908053410602 10432324

[B4] Cohen MS Chen YQ McCauley M Gamble T Hosseinipour MC Kumarasamy N Hakim JG Kumwenda J Grinsztejn B Pilotto JH Godbole SV Mehendale S Chariyalertsak S Santos BR Mayer KH Hoffman IF Eshleman SH Piwowar-Manning E Wang L Makhema J Mills LA De Bruyn G Sanne I Eron J Gallant J Havlir D Swindells S Ribaudo H Elharrar V Burns D Prevention of HIV-1 Infection with Early Antiretroviral Therapy N Engl J Med 2011 365 493 505 10.1056/NEJMoa1105243 21767103 PMC3200068

[B5] Tanser F Bärnighausen T Grapsa E Zaidi J Newell ML High coverage of ART associated with decline in risk of HIV acquisition in rural KwaZulu-Natal, South Africa Science 2013 339 966 971 10.1126/science.1228160 23430656 PMC4255272

[B6] The HIV Modelling Consortium Treatment as Prevention Editorial Writing Group HIV treatment as prevention: models, data, and questions–towards evidence-based decision-making PLoS Med 2012 9 e1001259 10.1371/journal.pmed.1001259 22802739 PMC3393655

[B7] Granich RM Gilks CF Dye C De Cock KM Williams BG Universal voluntary HIV testing with immediate antiretroviral therapy as a strategy for elimination of HIV transmission: a mathematical model Lancet 2009 373 48 57 10.1016/S0140-6736(08)61697-9 19038438

[B8] Dodd PJ Garnett GP Hallett TB Examining the promise of HIV elimination by ‘test and treat’ in hyperendemic settings AIDS 2010 24 729 735 10.1097/QAD.0b013e32833433fe 20154580 PMC2852517

[B9] Eaton JW Johnson LF Salomon JA Barnighausen T Bendavid E Bershteyn A Bloom DE Cambiano V Fraser C Hontelez JA Humair S Klein DJ Long EF Phillips AN Pretorius C Stover J Wenger EA Williams BG Hallett TB HIV treatment as prevention: systematic comparison of mathematical models of the potential impact of antiretroviral therapy on HIV incidence in South Africa PLoS Med 2012 9 e1001245 10.1371/journal.pmed.1001245 22802730 PMC3393664

[B10] Hontelez JAC Lurie MN Bärnighausen T Bakker R Baltussen R Tanser F Hallett TB Newell M-L De Vlas SJ Treatment as prevention for HIV in South Africa: different models show consistency in occurrence, but difference in timing of elimination and the overall impact of the intervention J Int AIDS Soc 2012 15 Suppl 3

[B11] Kitahata MM Gange SJ Abraham AG Merriman B Saag MS Justice AC Hogg RS Deeks SG Eron JJ Brooks JT Rourke SB Gill MJ Bosch RJ Martin JN Klein MB Jacobson LP Rodriguez B Sterling TR Kirk GD Napravnik S Rachlis AR Calzavara LM Horberg MA Silverberg MJ Gebo KA Goedert JJ Benson CA Collier AC Van Rompaey SE Crane HM Effect of early versus deferred antiretroviral therapy for HIV on survival N Engl J Med 2009 360 1815 1826 10.1056/NEJMoa0807252 19339714 PMC2854555

[B12] Sterne JA May M Costagliola D De Wolf F Phillips AN Harris R Funk MJ Geskus RB Gill J Dabis F Miro JM Justice AC Ledergerber B Fatkenheuer G Hogg RS Monforte AD Saag M Smith C Staszewski S Egger M Cole SR Timing of initiation of antiretroviral therapy in AIDS-free HIV-1-infected patients: a collaborative analysis of 18 HIV cohort studies Lancet 2009 373 1352 1363 19361855 10.1016/S0140-6736(09)60612-7 PMC2670965

[B13] Anglaret X Minga A Gabillard D Ouassa T Messou E Morris B Traore M Coulibaly A Freedberg KA Lewden C Menan H Abo Y Dakoury-Dogbo N Toure S Seyler C AIDS and non-AIDS morbidity and mortality across the spectrum of CD4 cell counts in HIV-infected adults before starting antiretroviral therapy in Cote d’Ivoire Clin Infect Dis 2012 54 714 723 10.1093/cid/cir898 22173233 PMC3275759

[B14] The ANRS 12249 study group ANRS 12249 TasP Protocol Version 1.2. 08/03/2012 ANRS, Paris [http://mereva.isped.u-bordeaux2.fr/tasp/html/tasp_documents/fichiers/ANRS12249_TasP-Protocol-V1.2-8mars2012-Signed.pdf]. Accessed 16 July 2013

[B15] Welz T Hosegood V Jaffar S Batzing-Feigenbaum J Herbst K Newell ML Continued very high prevalence of HIV infection in rural KwaZulu-Natal, South Africa: a population-based longitudinal study AIDS 2007 21 1467 1472 10.1097/QAD.0b013e3280ef6af2 17589193

[B16] Tanser F Barnighausen T Cooke GS Newell ML Localized spatial clustering of HIV infections in a widely disseminated rural South African epidemic Int J Epidemiol 2009 38 1008 1016 10.1093/ije/dyp148 19261659 PMC2720393

[B17] Zaidi J Grapsa E Tanser F Newell M Bärnighausen T HIV prevalence trends after scale-up of antiretroviral treatment: a population-based study in a poor rural community in KwaZulu-Natal J Int AIDS Soc 2012 15 Suppl 3

[B18] Barnighausen T Tanser F Newell ML Lack of a decline in HIV incidence in a rural community with high HIV prevalence in South Africa, 2003–2007 AIDS Res Hum Retroviruses 2009 25 405 409 10.1089/aid.2008.0211 19320571 PMC2853840

[B19] Barnighausen T Wallrauch C Welte A McWalter TA Mbizana N Viljoen J Graham N Tanser F Puren A Newell ML HIV incidence in rural South Africa: comparison of estimates from longitudinal surveillance and cross-sectional cBED assay testing PLoS One 2008 3 e3640 10.1371/journal.pone.0003640 18982059 PMC2572841

[B20] Tanser F Bärnighausen T Newell M Identification of Localized Clusters of High HIV Incidence in a Widely Disseminated Rural South African Epidemic: A Case for Targeted Intervention Strategies 2011 Boston, MA (USA): 18th Conference on Retroviruses and Opportunistic Infections (CROI) Abstract 137

[B21] Houlihan CF Bland RM Mutevedzi PC Lessells RJ Ndirangu J Thulare H Newell ML Cohort Profile: Hlabisa HIV Treatment and Care Programme Int J Epidemiol 2011 40 318 326 10.1093/ije/dyp402 20154009 PMC3195268

[B22] Maheswaran H Thulare H Stanistreet D Tanser F Newell ML Starting a home and mobile HIV testing service in a rural area of South Africa J Acquir Immune Defic Syndr 2012 59 e43 e46 10.1097/QAI.0b013e3182414ed7 22107821 PMC4239475

[B23] Tanser F Hosegood V Barnighausen T Herbst K Nyirenda M Muhwava W Newell C Viljoen J Mutevedzi T Newell ML Cohort Profile: Africa Centre Demographic Information System (ACDIS) and population-based HIV survey Int J Epidemiol 2008 37 956 962 10.1093/ije/dym211 17998242 PMC2557060

[B24] Tanser F Hosegood V Benzler J Solarsh G New approaches to spatially analyse primary health care usage patterns in rural South Africa Trop Med Int Health 2001 6 826 838 10.1046/j.1365-3156.2001.00794.x 11679131

[B25] Goldie SJ Yazdanpanah Y Losina E Weinstein MC Anglaret X Walensky RP Hsu HE Kimmel A Holmes C Kaplan JE Freedberg KA Cost-effectiveness of HIV treatment in resource-poor settings–the case of Cote d’Ivoire N Engl J Med 2006 355 1141 1153 10.1056/NEJMsa060247 16971720

[B26] Hontelez JA De Vlas SJ Tanser F Bakker R Barnighausen T Newell ML Baltussen R Lurie MN The impact of the new WHO antiretroviral treatment guidelines on HIV epidemic dynamics and cost in South Africa PLoS One 2011 6 e21919 10.1371/journal.pone.0021919 21799755 PMC3140490

[B27] Grosskurth H Mosha F Todd J Mwijarubi E Klokke A Senkoro K Mayaud P Changalucha J Nicoll A Ka-Gina G Newell J Mugeye K Mabey D Hayes R Impact of improved treatment of sexually transmitted diseases on HIV infection in rural Tanzania: randomised controlled trial Lancet 1995 346 530 536 10.1016/S0140-6736(95)91380-7 7658778

[B28] Tanser F Barnighausen T Hund L Garnett GP McGrath N Newell ML Effect of concurrent sexual partnerships on rate of new HIV infections in a high-prevalence, rural South African population: a cohort study Lancet 2011 378 247 255 10.1016/S0140-6736(11)60779-4 21763937 PMC3141142

[B29] World Health Organisation Antiretroviral therapy for HIV infection in adults and adolescents Recommendations for a public health approach (2010 revision) Geneva: World Health Organisation [http://whqlibdoc.who.int/publications/2010/9789241599764_eng.pdf]. Accessed18 January 2013 23741771

[B30] National Department of Health Clinical guidelines for the management of HIV/AIDS in adults and adolescents 2010 Republic of South Africa: Department of Health

[B31] Chaiyachati K Hirschhorn LR Tanser F Newell ML Barnighausen T Validating five questions of antiretroviral nonadherence in a public-sector treatment program in rural South Africa AIDS Patient Care STDS 2010 25 163 170 21269131 10.1089/apc.2010.0257 PMC3048836

[B32] Boyer S Clerc I Bonono CR Marcellin F Bile PC Ventelou B Non-adherence to antiretroviral treatment and unplanned treatment interruption among people living with HIV/AIDS in Cameroon: Individual and healthcare supply-related factors Soc Sci Med 2011 72 1383 1392 10.1016/j.socscimed.2011.02.030 21470734

[B33] Carrieri P Cailleton V Le Moing V Spire B Dellamonica P Bouvet E Raffi F Journot V Moatti JP The dynamic of adherence to highly active antiretroviral therapy: results from the French National APROCO cohort J Acquir Immune Defic Syndr 2001 28 232 239 11694829 10.1097/00042560-200111010-00005

[B34] Spire B Carrieri P Sopha P Protopopescu C Prak N Quillet C Ngeth C Ferradini L Delfraissy JF Laureillard D Adherence to antiretroviral therapy in patients enrolled in a comprehensive care program in Cambodia: a 24-month follow-up assessment Antivir Ther 2008 13 697 703 18771053

[B35] Duracinsky M Herrmann S Berzins B Armstrong AR Kohli R Le Coeur S Diouf A Fournier I Schechter M Chassany O The development of PROQOL-HIV: an international instrument to assess the health-related quality of life of persons living with HIV/AIDS J Acquir Immune Defic Syndr 2012 59 498 505 10.1097/QAI.0b013e318245cafe 22205438

[B36] Duracinsky M Lalanne C Le Coeur S Herrmann S Berzins B Armstrong AR Lau JT Fournier I Chassany O Psychometric validation of the PROQOL-HIV questionnaire, a new health-related quality of life instrument-specific to HIV disease J Acquir Immune Defic Syndr 2012 59 506 515 10.1097/QAI.0b013e31824be3f2 22293550

[B37] Holzemer WL Uys LR Chirwa ML Greeff M Makoae LN Kohi TW Dlamini PS Stewart AL Mullan J Phetlhu RD Wantland D Durrheim K Validation of the HIV/AIDS Stigma Instrument - PLWA (HASI-P) AIDS Care 2007 19 1002 1012 10.1080/09540120701245999 17851997

[B38] Ukoumunne OC Carlin JB Gulliford MC A simulation study of odds ratio estimation for binary outcomes from cluster randomized trials Stat Med 2007 26 3415 3428 17154246 10.1002/sim.2769

[B39] Mancl LA DeRouen TA A covariance estimator for GEE with improved small-sample properties Biometrics 2001 57 126 134 10.1111/j.0006-341X.2001.00126.x 11252587

[B40] Attia S Egger M Muller M Zwahlen M Low N Sexual transmission of HIV according to viral load and antiretroviral therapy: systematic review and meta-analysis Aids 2009 23 11 1397 1404 10.1097/QAD.0b013e32832b7dca 19381076

[B41] Anglemyer A Rutherford GW Baggaley RC Egger M Siegfried N Antiretroviral therapy for prevention of HIV transmission in HIV-discordant couples Cochrane Database Syst Rev 2011 8 CD009153 21833973 10.1002/14651858.CD009153.pub2

[B42] World Health Organisation Guidance on Couples HIV Testing and Counselling including Antiretroviral Therapy for Treatment and Prevention in Serodiscordant Couples Recommendations for a public health approach 2012 Geneva: World Health Organisation In 23700649

[B43] World Health Organisation Consolidated guidelines on the use of antiretroviral drugs for treating and preventing HIV infection Recommendations for a public health approach 2013 Geneva: World Health Organisation In 24716260

[B44] Chi BH Cantrell RA Zulu I Mulenga LB Levy JW Tambatamba BC Reid S Mwango A Mwinga A Bulterys M Adherence to first-line antiretroviral therapy affects non-virologic outcomes among patients on treatment for more than 12 months in Lusaka, Zambia Int J Epidemiol 2009 38 3 746 756 10.1093/ije/dyp004 19223334 PMC2689395

[B45] Maqutu D Zewotir T North D Naidoo K Grobler A Factors affecting first-month adherence to antiretroviral therapy among HIV-positive adults in South Africa Afr J AIDS Res 2010 9 2 117 124 10.2989/16085906.2010.517478 21779200 PMC3137932

[B46] Uy J Armon C Buchacz K Wood K Brooks JT Initiation of HAART at higher CD4 cell counts is associated with a lower frequency of antiretroviral drug resistance mutations at virologic failure J Acquir Immune Defic Syndr 2009 51 4 450 453 10.1097/QAI.0b013e3181acb630 19474757

[B47] Barnighausen T Bloom DE Humair S Economics of antiretroviral treatment vs. circumcision for HIV prevention Proceedings of the National Academy of Sciences of the United States of America 2012 109 52 21271 21276 10.1073/pnas.1209017110 23223563 PMC3535659

