# Supplementary material for: Evaluation of the impact of immediate versus WHO recommendations-guided antiretroviral therapy initiation on HIV incidence: the ANRS 12249 TasP (Treatment as Prevention) trial in Hlabisa sub-district, KwaZulu-Natal, South Africa: study protocol for a cluster randomised controlled trial
Source: Trials. 2013 Jul 23;14:230. doi: 10.1186/1745-6215-14-230 (PMC3750830; doi:10.1186/1745-6215-14-230)
Supplement: Additional file 1 Table S1 — Composition of the TasP Study Group. [file 1745-6215-14-230-S1.docx]

**Table S1: Composition of the TasP Study Group**

|  | **Name** | **Affiliation** |
| --- | --- | --- |
| **Investigators** |  |  |
| Co-PI (France) | François Dabis | - Univ. Bordeaux, ISPED, Centre Inserm U897- Epidemiologie-Biostatistique, F-33000 Bordeaux, France |
|  |  | - INSERM, ISPED, Centre Inserm U897- Epidemiologie-Biostatistique, F-33000 Bordeaux, France |
| Co-PI (South Africa) | Marie-Louise Newell | -Africa Centre for Health and Population Studies, University of KwaZulu-Natal, South Africa  -Faculty of Medicine, University of Southampton, UK |
| **Coordinators** |  |  |
| Trial Coordinator and HIV Clinician (South Africa) | Collins Iwuji | -Africa Centre for Health and Population Studies, University of KwaZulu-Natal, South Africa  -Dept of Sexual Health & HIV Medicine, Brighton and Sussex University Hospitals NHS Trust, UK |
| Trial Coordinator (France) | Joanna Orne-Gliemann | - Univ. Bordeaux, ISPED, Centre Inserm U897- Epidemiologie-Biostatistique, F-33000 Bordeaux, France |
|  |  | - INSERM, ISPED, Centre Inserm U897- Epidemiologie-Biostatistique, F-33000 Bordeaux, France |
| **Team in South Africa** |  |  |
| Nurse manager | Nonhlanhla Okesola | Africa Centre for Health and Population Studies, University of KwaZulu-Natal, South Africa |
| Social sciences | John Imrie | - Africa Centre for Health and Population Studies, University of KwaZulu-Natal, South Africa |
|  |  | - Centre for Sexual Health and HIV Research, Research Department of Infection and Population, Faculty of Population Health Sciences, University College London, London, UK |
| Health economics | Till Bärnighausen | -Africa Centre for Health and Population Studies, University of KwaZulu-Natal, South Africa |
|  |  | -Dept of Global Health & Population, Harvard School of Public Health, Harvard Univ. Boston |
| Clinical science | Ruth Bland | Africa Centre for Health and Population Studies, University of KwaZulu-Natal, South Africa |
|  | Richard Lessells | -Africa Centre for Health and Population Studies, University of KwaZulu-Natal, South Africa |
|  |  | -Dept of Clinical Research, London School of Hygiene and Tropical Medicine, UK |
| Epidemiology and Biostatistics | Frank Tanser | Africa Centre for Health and Population Studies, University of KwaZulu-Natal, South Africa |
| Bioinformatics | Tulio de Oliveira | Africa Centre for Health and Population Studies, University of KwaZulu-Natal, South Africa |
| Virology | Johannes Viljoen | Africa Centre for Health and Population Studies, University of KwaZulu-Natal, South Africa |
| Data management | Colin Newell | Africa Centre for Health and Population Studies, University of KwaZulu-Natal, South Africa |
| Treatment programme | Kevi Naidu | Africa Centre for Health and Population Studies, University of KwaZulu-Natal, South Africa |
| **Team in France, Switzerland, and USA** |  |  |
| Social Sciences | France Lert | INSERM U1018, CESP, Epidemiology of Occupational and Social Determinants of Health, Villejuif, France |
|  |  | University of Versailles Saint-Quentin, UMRS 1018, Villejuif, France |
|  | Rosemary Dray-Spira | INSERM U1018, CESP, Epidemiology of Occupational and Social Determinants of Health, Villejuif, France |
|  |  | University of Versailles Saint-Quentin, UMRS 1018, Villejuif, France |
|  | Joseph Larmarange | - CEPED (Centre Population & Développement-UMR 196-Paris Descartes/INED/IRD), IRD (Institut de Recherche pour le Développement), Paris, France. |
|  |  | - Africa Centre for Health and Population Studies, University of KwaZulu-Natal, South Africa |
| Health economics | Bruno Spire | - INSERM, UMR912 (SESSTIM), 13006, Marseille, France |
|  |  | - Aix Marseille Université, UMR_S912, IRD, 13006, Marseille, France |
|  |  | - ORS PACA, Observatoire Régional de la Santé Provence-Alpes-Côte d’Azur, 13006, Marseille, France |
|  | Sylvie Boyer | - INSERM, UMR912 (SESSTIM), 13006, Marseille, France |
|  |  | - Aix Marseille Université, UMR_S912, IRD, 13006, Marseille, France |
|  |  | - ORS PACA, Observatoire Régional de la Santé Provence-Alpes-Côte d’Azur, 13006, Marseille, France |
| Adult Medicine | Alexandra Calmy | Service des maladies infectieuses, Hôpital Universitaire de Geneve, 1211 Genève. |
| Virology | Marie-Laure Chaix | EA 3620, Université Paris-Descartes, Laboratoire de Virologie, Hôpital Necker-Enfants Malades, AP-HP, Paris |
| Data management | Sophie Karcher | - Univ. Bordeaux, ISPED, Centre Inserm U897- Epidemiologie-Biostatistique, F-33000 Bordeaux, France |
|  |  | - INSERM, ISPED, Centre Inserm U897- Epidemiologie-Biostatistique, F-33000 Bordeaux, France |
| Statistician | Rodolphe Thiébaut | - Univ. Bordeaux, ISPED, Centre Inserm U897- Epidemiologie-Biostatistique, F-33000 Bordeaux, France |
|  |  | - INSERM, ISPED, Centre Inserm U897- Epidemiologie-Biostatistique, F-33000 Bordeaux, France |
| Modelling | Kenneth Freedberg | Massachusetts General Hospital, Harvard Medical School, Boston, MA, USA. |

**Scientific advisory board**

-Chair: Bernard Hirschel (Switzerland)

-International experts: Xavier Anglaret (Abidjan), Hoosen Cooavdia (South Africa), Bruno Giraudeau (France), Jean-Michel Molina (France), Lynn Morris (South Africa), François Venter (South Africa), Sibongile Zungu (South Africa)

-Community representatives: Eric Fleutelot (France), Eric Goemaere (South Africa), Calice Talom (Cameroon)

-Study members: Till Bärnighausen, Alexandra Calmy, François Dabis, John Imrie, Collins Iwuji, Marie-Louise Newell, Joanna Orne-Gliemann, Bruno Spire, Frank Tanser

-Sponsor representatives (ANRS): Brigitte Bazin, Alpha Diallo, Claire Rekacewicz,

-Pharmaceutical company representatives: Alix De Jaquelot (MSD), Alice Jacob (Gilead)

**Data safety and monitoring board**

-Chair: Patrick Yeni (France)

-Members: Sinead Delany-Moretlwe (South Africa), Nathan Ford (South Africa), Catherine Hankins (Netherlands), Hakima Himmich (Morocco), Helen Weiss (UK),
